# Cancer survival analysis using semi-supervised learning method based on Cox and AFT models with L_1/2_ regularization

**DOI:** 10.1186/s12920-016-0169-6

**Published:** 2016-03-01

**Authors:** Yong Liang, Hua Chai, Xiao-Ying Liu, Zong-Ben Xu, Hai Zhang, Kwong-Sak Leung

**Affiliations:** 1 State Key Laboratory of Quality Research in Chinese Medicines & Faculty of Information Technology, Macau University of Science and Technology, Macau, China; 2 Faculty of Science, Xi’an Jiaotong University, Xi’an, China; 3 Department of Computer Science and Engineering, The Chinese University of HongKong, HongKong, China

**Keywords:** Cancer survival analysis, Semi-supervised learning, Gene selection, Regularization, Cox proportional hazards model, Accelerated failure time model

## Abstract

**Background:**

One of the most important objectives of the clinical cancer research is to diagnose cancer more accurately based on the patients’ gene expression profiles. Both Cox proportional hazards model (Cox) and accelerated failure time model (AFT) have been widely adopted to the high risk and low risk classification or survival time prediction for the patients’ clinical treatment. Nevertheless, two main dilemmas limit the accuracy of these prediction methods. One is that the small sample size and censored data remain a bottleneck for training robust and accurate Cox classification model. In addition to that, similar phenotype tumours and prognoses are actually completely different diseases at the genotype and molecular level. Thus, the utility of the AFT model for the survival time prediction is limited when such biological differences of the diseases have not been previously identified.

**Methods:**

To try to overcome these two main dilemmas, we proposed a novel semi-supervised learning method based on the Cox and AFT models to accurately predict the treatment risk and the survival time of the patients. Moreover, we adopted the efficient L_1/2_ regularization approach in the semi-supervised learning method to select the relevant genes, which are significantly associated with the disease.

**Results:**

The results of the simulation experiments show that the semi-supervised learning model can significant improve the predictive performance of Cox and AFT models in survival analysis. The proposed procedures have been successfully applied to four real microarray gene expression and artificial evaluation datasets.

**Conclusions:**

The advantages of our proposed semi-supervised learning method include: 1) significantly increase the available training samples from censored data; 2) high capability for identifying the survival risk classes of patient in Cox model; 3) high predictive accuracy for patients’ survival time in AFT model; 4) strong capability of the relevant biomarker selection. Consequently, our proposed semi-supervised learning model is one more appropriate tool for survival analysis in clinical cancer research.

## Background

An important objective of clinical cancer research is to develop tools to accurately predict the survival time and risk profile of patients based on the DNA microarray data and various clinical parameters. There are several existing techniques in the literature for performing this type of survival analysis. Among of them, both Cox proportional hazards model (Cox) [1] and the accelerated failure time model (AFT) [2] have been widely used. Cox model is the most popular approach by far in survival analysis to assess the significance of various genes in the survival risk of patients through the hazard function. On the other hand, the requirement for analyzing failure time data arises in investigating the relationship between a censored survival outcome and high-dimensional microarray gene expression profiles. Therefore, AFT model has been studied extensively in recent years. However, various current cancer survival analysis mechanisms have not demonstrated themselves to be very accurate as expected. The accuracy problems, in essence, are related to some fundamental dilemmas in cancer survival analysis. We believe any attempt to improve the accuracy of survival analysis method has to compromise between these two dilemmas:

- The small sample size and censored survival data versus high dimensional covariates dilemma in Cox model

High-dimensional survival analysis in particular has attracted much interest due to the popularity of microarray studies involving survival data. This is statistically challenging because the number of genes, *p*, is typically hundreds of times larger than the number of microarray samples, *n* (*p*> > *n*). For survival analysis, sample size is reduced significantly by the availability of follow-up data for the analyzed samples. In fact, in publicly available gene expression databases, only a small fraction of human-tumor microarray datasets provides clinical follow-up data. A “low-risk” or “high-risk” classification based on Cox model usually relies on traditional supervised learning techniques, in which only completed data (i.e., data from samples with clinical follow-up) can be used for learning, while censored data (i.e., data from samples without clinical follow-up) are disregarded. Thus, the small sample size and censored survival data remain a bottleneck in obtaining robust and accurate classifiers with Cox model. Recently a technique called semi-supervised learning [3] in machine learning suggests that censored data, when used in conjunction with limited amount of completed data, can produce considerable improvement in learning accuracy. Indeed, semi-supervised learning has been proved to be effective in solving different biological problems, such as protein classification [4, 5], drug-protein interaction prediction [6] and prediction of interactions between disease and human proteins [7]. Moreover, there are some semi-supervised learning approaches worked on the gene expression data. For example, “corrected” Cox scores were used for semi-supervised prediction using principal component regression by Bair and Tibshirani [8] and the semi-supervised classification using nearest-neighbor shrunken centroid clustering by Tibshirani et al. [9].

- The similar phenotype disease versus different genotype cancer dilemma in the AFT model

In the accelerated failure time model, to increase the available sample size and get the more accurate result, each censored observation time is replaced with the imputed value using some estimators, such as the inverse probability weighting (IPW) [10] method, mean imputation method, Buckley-James method [11] and rank-based method. In fact, these estimation methods assume that the AFT model was used for the patients with similar phenotype cancer, and the survival times should satisfy the same unspecified common probability distribution. Nevertheless, the disparity we see in disease progression and treatment response can be attributed to that the similar phenotype cancer may be completely different diseases on the molecular genotype level. So we need to identify different cancer genotypes. Can we do it based exclusively on the clinical data? For example, patients can be assigned to a “low-risk” or a “high-risk” subgroup based on whether they were still alive or whether their tumour had metastasized after a certain amount of time. This approach has also been used to develop procedures to diagnose patients [12]. However, by dividing the patients into subgroups just based on their survival times, the resulting subgroups may not be biologically meaningful. Suppose, for example, the underlying cell types of each patient are unknown. If we were to assign patients to “low-risk” and “high-risk” subgroups based on their survival times, many patients would be assigned to the wrong subgroup, and any future predictions based on this model would be suspect. Therefore, we need propose more accurate classification methods by identifying these underlying cancer subtypes based on microarray data and clinical data together, and build a model that can determine which subtype is present in future patients.

Our idea in this study is to strike a tactical balance between the two contradictory dilemmas. We propose a novel semi-supervised learning method based on the combination of Cox and AFT models with L_1/2_ regularization for high-dimensional and low sample size biological data. In our semi-supervised learning framework, the Cox model can classify the “low-risk” or a “high-risk” subgroup though samples as many as possible to improve its predictive accuracy. Meanwhile, the AFT model can estimate the censored data in the subgroup, in which the samples have the same molecular genotype.

## Methods

### Cox proportional hazards model (Cox)

The Cox proportional hazards model is now the most widely used for survival analysis to classify the patients into “low-risk” or “high-risk” subgroup after prognostic. Under the Cox model, the hazard function for the covariate matrix x with sample size n and the number of genes p is specified as ***λ***(***t***) = ***λ***_0_(***t***)exp(***β***′***x***), where *t* is the survival time and the baseline hazard function λ_0_(t) is common to all subjects, but is unspecified or unknown. Let ordered risk set at time t(r) be denoted by *Rr = {j∈1,…, n:tj ≥ t(r)}*. Assume that censoring is non informative and that there are no tied event times. The Cox log partial likelihood can then be defined as

1 $$ l\left(\beta \right)=\frac{1}{\mathrm{n}}{\displaystyle {\sum}_{\mathrm{r}\in \mathrm{D}}} \ln \left(\frac{ \exp \left({\upbeta^{\prime}\mathrm{x}}_{\left(\mathrm{r}\right)}\right)}{{\displaystyle {\sum}_{\mathrm{j}\in {\mathrm{R}}_{\mathrm{r}}}} \exp \left({\upbeta}^{\prime }{\mathrm{x}}_{\mathrm{j}}\right)}\right) $$

Where *D* denotes the set of indices for observed events.

### Accelerated failure time model (AFT)

The AFT model is a linear regression model for survival analysis, in which the logarithm of response t_i_ is related linearly to covariates x_i_:

2 $$ h\left({t}_i\right)={\beta}_0+{x_i}^{\prime}\beta +{\varepsilon}_i,\kern0.75em i=1,\dots, n, $$

where h(.) is the log transformation or some other monotone function. In this case, the Cox assumption of multiplicative effect on hazard function is replaced with the assumption of multiplicative effect on outcome. In other words, it is assumed that the variables *x*_*i*_ act multiplicatively on time and therefore affect the rate at which individual *i* proceeds along the time axis. Because censoring is present, the standard least squares approach cannot be employed to estimate the regression parameters in Eq. (2) even when *p* < *n*.

One approach for AFT model implementation entails the replacement of censored *t*_*i*_ with imputed values. In order to simplify the method, we use Kaplan-Meier weight approach to estimate the censored data in the least square criterion. Since for high dimensional and low simple size data, the Kaplan-Meier weight estimator is more efficient than the Buckley-James and rank based approaches. Moreover, it also has rigorously and strong theoretical justifications under reasonable conditions [13]. For each censored t_*i*_ with the conditional expectation of *t*_*j*_ given *t*_*j*_ > *t*_*i*_ [14], the imputed value *h*(*t*_*i*_) can then be given by

3 $$ h\left({t}_i^{*}\right)=\left({\delta}_i\right)h\left({t}_i\right)+\left(1-{\delta}_i\right){\left\{\widehat{S}\left({t}_i\right)\right\}}^{-1}{\displaystyle {\sum}_{t_{(r)>}{t}_i}}h\left({t}_{(r)}\right)\varDelta \widehat{S}\left({t}_{(r)}\right), $$

where *Ŝ* is the Kaplan-Meier estimator (Kaplan and Meier, 1958) of the survival function and Δ*Ŝ*(*t*_(*r*)_) is the step of *Ŝ* at time *t*_(*r*)_ [15].

### L_1/2_ regularization

In recent years, various regularization methods for survival analysis under the Cox and AFT models have been proposed, which perform both continuous shrinkage and automatic gene selection simultaneously. For example, Cox-based methods utilizing kernel transformations [16], threshold gradient descent minimization [17], and lasso penalization [18] have been proposed. Likewise, a few authors have proposed variable selection methods based on accelerated failure time models. Most of these procedures are based on L_1_ -norm, however, the results of L_1_ regularization are not good enough for spartity, especially in biology research. Theoretically, the L_q_ (0 < q < 1) type regularization with the lower value of q would lead to better solutions with more sparsity. Moreover, among L_q_ regularizations with q ∈ (0, 1), only L_1/2_ and L_2/3_ regularizations permit an analytically expressive thresholding representation [19]. In the literature [19], Xu et al. investigated that when 0 < q < 1/2, there are not obvious difference in the variable selection performance of L_q_ (0 < q < 1/2) regularization, but solving the L_1/2_ regularization is much efficient compared to the L_0_ regularization. On the other hand, the L_1/2_ regularization can yield most sparse solutions among L_q_ (1/2 < q < 1) regularizations. Moreover, they also proved some attractive properties of the L_1/2_ regularization, such as unbiasedness, sparsity and oracle properties. Our previous works have also demonstrated the efficiencies of L_1/2_ regularization for Cox and AFT models respectively [20]. The sparse L_1/2_ regularization model has expressed as:

4 $$ \upbeta =\mathrm{argmin}\left\{l\left(\beta \right)+\lambda {\displaystyle \sum_{j=1}^p}{\left|{\beta}_j\right|}^{1/2}\right\} $$

where *l* is loss function and λ is tuning parameter. Since the penalty function of L_1/2_ regularization is nonconvex, which raises numerical challenges in fitting the Cox and AFT models. Recently, coordinate descent algorithms [21] for solving nonconvex regularization approach (such as SCAD, MCP) have been shown significantly efficiency and convergence [22]. The algorithms optimize a target function with respect to a single parameter at a time, iteratively cycling through all parameters until reached its convergence. Since the computational burden increases only linearly with the number of the covariates *p*, coordinate descent algorithms can be a powerful tool for solving high-dimensional problems.

Therefore, in this paper, we introduce a novel univariate half thresholding operator of the coordinate descent algorithm for the L_1/2_ regularization, which can be expressed as:

5 $$ \begin{array}{l}{\beta}_j= New\_ Half\left({\omega}_j,\lambda \right)=\left\{\frac{2}{3}\right.{\omega}_j\left(1+ \cos \left(\frac{2\left(\pi -{\varphi}_{\lambda}\left({\omega}_j\right)\right)}{3}\right)\right)\kern1em  if\left|{\omega}_j\right|>\frac{\sqrt[3]{54}}{4}{\left(\lambda \right)}^{\frac{2}{3}}\\ {}\kern1em 0\kern9em  otherwise\end{array} $$

where *ỹ*_*i*_^(*j*)^ = ∑_*k* ≠ *j*_*x*_*ik*_*β*_*k*_ as the partial residual for fitting *β*_*j*_*, ω*_*j*_ = ∑_*i* = 1_^*n*^*x*_*ij*_(*y*_*i*_ − *ỹ*_*i*_^(*j*)^), and ${\varphi}_{\lambda}\left(\omega \right)= arccos\Big(\frac{\lambda }{8}{\left(\frac{\left|\omega \right|}{3}\right)}^{-\frac{3}{2}}$.

*Remark*: In our previous work [23], we used $\frac{3}{4}{\left(\uplambda \right)}^{\frac{2}{3}}$ for represent L_1/2_ regularization thresholding operator. Here, we introduced a new half thresholding representation $\frac{\sqrt[3]{54}}{4}{\left(\uplambda \right)}^{\frac{2}{3}}$. This new value is more precisely and effectively than the old one. Since it is known that the quantity of the solutions of a regularization problem depends seriously on the setting of the regularization parameter λ. Based on this novel thresholding operator, when λ is chosen by some efficient parameters tuning strategy, such as cross-validation, the convergence of the algorithm is proved [24].

### Our proposed semi-supervised learning method

**Fig. 1:**
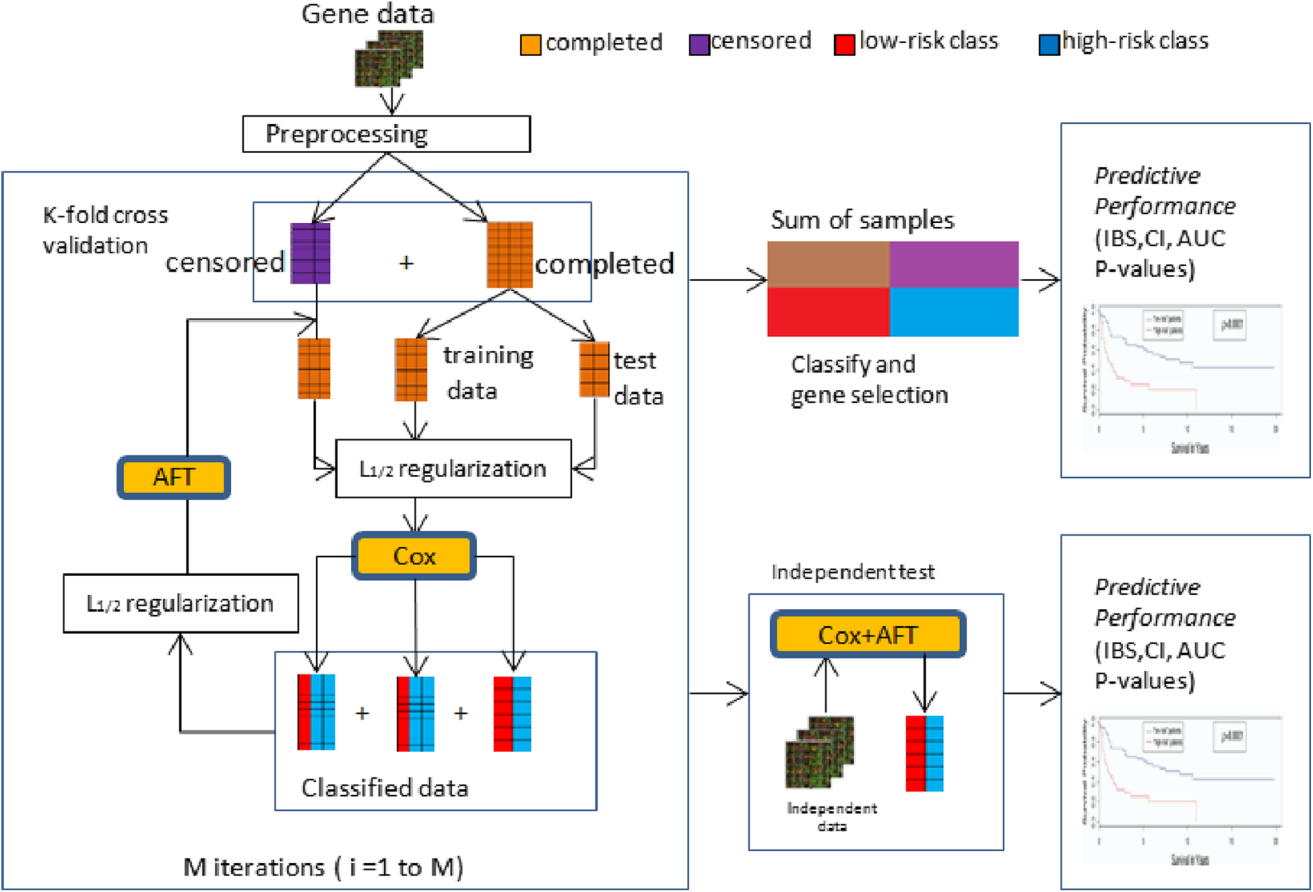
Workflow for the development and evaluation of the semi-supervised learning framework for survival analysis

Figure 1 illustrates the overview of our proposed semi-supervised learning development and evaluation workflow. Microarray gene expression data on a specific cancer type are collected, processed, and separated into completed samples and censored samples. In order to identify tumor subclasses that were both biologically meaningful and clinically relevant, we applied the L_1/2_ regularized Cox model on the completed data to select a group of outcome-related genes firstly. Thus, all samples including completed and censored cases can be subsequently classified into “low-risk” and “high-risk” classes. Once such classes are identified, we can evaluate the censored data using the mean imputation approach based on the completed data belonged to the same risk classes, because they are correlated to similar disease biologically meaningful at the molecular level. When the censored data replaced by the appropriate imputation values, the L_1/2_ regularized AFT model can be used to select a list of genes that correlate with the clinical variable of interest, and reevaluate the censored data based on these selected genes. A stratified *K*-fold cross-validation is used for regularization parameter tuning. We repeated this semi-supervised learning procedure including Cox and AFT steps multiple time with increasing number of available training data and estimating the censored data based on the similar genotype disease.

In the semi-supervised learning framework, the predictive accuracy of the Cox and AFT models would be improved because the number of the training data increased and the censored data were imputed reasonably. The L_1/2_ regularization approach can select the significant relevant gene sets based on the Cox and AFT models respectively.

In our proposed semi-supervised learning method, the censored data are evaluated from the same risk class to improve prediction performance. However, there are some observable errors in the imputations of the censored data. For example, the estimated survival time by AFT model was even less than the censored time. We regarded them as error estimations, and would not use them for model training.

In this paper, two parameters were used to test the performances obtained by different methods.

### Integrated Brier-Score (IBS)

The Brier Score (BS) [25] is defined as a function of time *t* > 0 by:

6 $$ BS(t)=\frac{1}{n}{\displaystyle \sum_{i=1}^n\left[\frac{\widehat{S}{\left(t\Big|{X}_i\right)}^21\left({t}_i\le t\wedge {\delta}_i=1\right)}{\widehat{G}\left({t}_i\right)}+\frac{{\left(1-\widehat{S}\left(t\Big|{X}_i\right)\right)}^21\left({t}_i>t\right)}{\widehat{G}(t)}\right]} $$

where *Ĝ*(⋅) denotes the Kaplan-Meier estimation of the censoring distribution and *Ŝ*(⋅|*X*_*i*_) stands to estimate survival for the patient *i*. Note that the *BS*(*t*) is dependent on the time *t*, and its values are between 0 and 1. The good predictions at the time *t* result in small values of BS. The integrated Brier Score (IBS) is given by:

7 $$ IBS=\frac{1}{ \max \left({t}_i\right)}{\displaystyle \underset{0}{\overset{ \max \left({t}_i\right)}{\int }}BS(t)dt} $$

The IBS is used to assess the goodness of the predicted survival functions of all observations at every time between 0 and max(*t*_*i*_).

### Concordance Index (CI)

The Concordance Index (CI) can be interpreted as the fraction of all pairs of subjects which predicted survival times are correctly ordered among all subjects that can actually be ordered. By the CI definition, we can determine *t*_*i*_ > *t*_*j*_ when *f*_*i*_ > *f*_*j*_ and *δ*_j_ = 1 where *f*(⋅) is survival function. The pairs for which neither *t*_*i*_ > *t*_*j*_ nor *t*_*i*<_*t*_*j*_can be determined are excluded from the calculation of CI. Thus, the CI is defined as:

8 $$ CI=\frac{{\displaystyle \sum_i{\displaystyle \sum_j1\left({f}_i<{f}_j\wedge {\delta}_i=1\right)}}}{{\displaystyle \sum_i{\displaystyle \sum_j1\left({t}_i<{t}_j\wedge {\delta}_i=1\right)}}} $$

Note that the values of CI are between 0 and 1, the perfect predictions of the building model would lead to 1 while have a CI of 0.5 at random.

## Results

### Simulated experiment

We adopted the simulation scheme in R. Bender’s work [26]. The generation procedure of the simulated data is as follows:

Step 1: we generate γ_i0_, γ_i1_,…, γ_ip_ (i = 1,…,n) independently from standard normal distribution and set: ${X}_{ij}={\gamma}_{ij}\sqrt{1-\rho }+{\gamma}_{i0}\sqrt{\rho }$ (j = 1,…, p) where ρ is the correlation coefficient.
Step 2: The survival time y_i_ is written as: ${\mathrm{y}}_{\mathrm{i}}=\frac{1}{\alpha } log\left(1-\frac{\alpha * \log (U)}{\omega * \exp \left(\beta X\right)}\right)$ which U is an uniformly distributed variable, ω is the scale parameter, α is the shape parameter.
Step 3: Censoring time point y_i_′ (i = 1,…n) is obtained from an random distribution E (θ), where θ is determined by specify censoring rate.
Step 4: Here we define y_i_ = min(y_i_, y_i_′) and δ_i_ = I(y_i_ < y_i_′), the observed data represented as (y_i_, x_i_, δ_i_) for the model are generated.

In our simulated experiments, we build high-dimensional and low sample size datasets. In every dataset, the dimension of the predictive genes is *p* = 1000, in which 10 prognostic genes and their corresponding coefficients are nonzero. The coefficients of the remaining 990 genes are zero. About 40 % of the data in each subgroup are right censored. We considered the training sample sizes are *n* = 100, 200, 300 and the correlation coefficients of genes areρ = 0 and ρ = 0.3 respectively. The simulated data were applied to the single Cox, single AFT and semi-supervised learning approach with Cox and AFT models. For gene selection, we use L_1/2_ regularization approach and the regularization parameters are tuned by 5-fold cross validation. To assess the variability of the experiment, each method is evaluated on a test set including 200 samples, and replicated over 50 random training and test partitions.

**Fig. 2:**
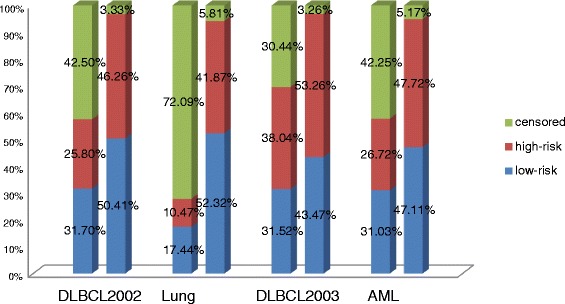
The percentage of different types of samples in original datasets and the datasets processed by our semi-supervised learning approach

Figure 2 shows the percentage of data distribution processed by our semi-supervised learning model with L_1/2_ regularization in different parameter settings (a: *n* = 100, ρ = 0.3; b: *n* = 100, ρ = 0; c: *n* = 200, ρ = 0.3; d: *n* = 200, ρ = 0; e: *n* = 300, ρ = 0.3; f: *n* = 300, ρ = 0;). The first cylinder represents the simulated dataset, and the cylinders *a-f* present the form of the dataset processed by our semi-supervised learning model. Compared to the original dataset, the most censored data can be reasonable estimated to the available data by semi-supervised learning model. For example, when the training sample *n* = 300 and the correlation coefficientρ = 0, just 2.41 % censored data cannot conjugate into the available samples because their imputed survival time based on the AFT model is smaller than their observed censored time. Moreover, we can see that with the sample size increases or the correction coefficient decreases, more censored data can be correctly estimated to available training data.

**Fig. 3:**
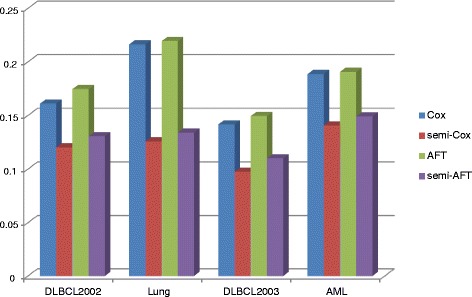
The IBS obtained by the Cox and AFT models with and without semi-supervised learning approach for the four gene expression datasets

The classification accuracy under the correlation coefficient ρ = 0.3 with different training sample size setting was demonstrated in Fig. 3, the sum of red and blue part represent the samples which can be correctly classified by the Cox model. The first cylinder in each group represents the result obtained by Cox model, and the second one represents the result obtained by our semi-supervised learning model. No matter in which group, the semi-supervised learning model obtained the high improvements of the classification performance. When the training sample size *n* = 100, 200, 300, more than 32.23, 20.55 and 15.63 % samples were correctly classified by semi-Cox model when comparing with the results of the single Cox model.

The precision of our semi-supervised learning model with L_1/2_ regularization was given in Table 1. The precision is got from the number of correct selected genes divided the total number of selected genes by the methods. With the sample size increase or the correction coefficients of the features decrease, the classification performances of each model become better. We found the single Cox and single AFT model is difficult to select the whole correct genes in the dataset. This means these models selected too few corrected genes and many other irrelevant genes in their results. This made their prediction precision very low. Nevertheless, our semi-supervised learning model solves this problem, the precisions of the semi-Cox or the semi-AFT were both higher than that obtained by the single Cox or single AFT model. After processed by our semi supervised learning method, the number of selected correct genes was increased, and the number of total selected genes were decreased, the semi-Cox achieved about 130 % improvements in precision compared to the single Cox model. Although the precision improvement of semi-AFT model is smaller than that of the semi-Cox model, it can select most correct genes under different parameter settings. Therefore we think our semi-supervised learning method can significantly improve the accuracy of prediction for survival analyses with the high-dimensional and low sample size gene expression data.

**Table 1:** The performance of the Cox and AFT models with and without the semi-supervised learning approach in simulated experiment (the average numbers and the standard deviations (in brackets) were listed in 50 runs)

Cor.	Size	Cox			Semi-Cox		
		Correct	Selected	Precision	Correct	Selected	Precision
	100	4.06 (1.39)	24.44 (4.65)	0.166 (0.044)	6.58 (1.41)	16.96 (6.41)	0.388 (0.080)
*ρ* = 0	200	5.62 (1.64)	28.22 (6.16)	0.199 (0.031)	8.68 (1.56)	17.84 (5.72)	0.487 (0.078)
	300	8.02 (1.43)	35.18 (5.81)	0.228 (0.029)	9.76 (0.98)	19.02 (5.41)	0.513 (0.087)
	100	3.90 (1.43)	24.38 (5.83)	0.159 (0.041)	6.46 (1.37)	17.08 (6.05)	0.378 (0.075)
*ρ* = 0.3	200	5.68 (1.42)	29.64 (6.19)	0.192 (0.035)	8.62 (1.11)	17.86 (5.45)	0.483 (0.074)
	300	7.84 (1.55)	35.86 (5.96)	0.219 (0.037)	9.42 (0.68)	18.54 (5.10)	0.508 (0.082)
Cor.	Size	AFT	Semi-AFT				
		Correct	Selected	Precision	Correct	Selected	Precision
	100	5.02 (1.61)	38.74 (6.27)	0.130 (0.029)	6.84 (1.37)	35.52 (6.17)	0.192 (0.031)
*ρ* = 0	200	7.12 (1.30)	46.68 (6.03)	0.152 (0.025)	8.84 (1.18)	42.16 (5.38)	0.210 (0.039)
	300	8.90 (0.99)	56.54 (6.85)	0.157 (0.019)	9.86 (0.46)	50.84 (5.49)	0.194 (0.027)
	100	4.74 (1.19)	39.54 (5.88)	0.120 (0.030)	6.72 (1.43)	35.84 (6.43)	0.188 (0.033)
*ρ* = 0.3	200	6.98 (1.50)	47.02 (6.32)	0.148 (0.024)	8.78 (1.02)	44.96 (6.95)	0.195 (0.031)
	300	8.80 (1.02)	56.82 (6.30)	0.155 (0.022)	9.78 (0.50)	49.31 (5.86)	0.198 (0.034)

### Simulation analysis of real microarray datasets

In this section, the proposed semi-supervised learning approach was applied to the four real gene expression datasets respectively, such as DLBCL (2002) [27], DLBCL (2003) [28], Lung cancer [29], AML [30]. The brief information of these datasets is summarized in Table 2.

**Table 2:** The detail information of four real gene expression datasets used in the experiments

Datasets	No. of genes	No. of samples	No. of censored
DLBCL (2002)	7399	240	102
DLBCL (2003)	8810	92	28
Lung cancer	7129	86	62
AML	6283	116	49

In order to accurately assess the performance of the semi-supervised learning approach, the real datasets were randomly divided into two pieces: two thirds of the available patient samples, which include the completed and correct imputed censored data, were put in the training set used for estimation and the remaining completed and censored patients’ data would be used to test the prediction capability. We used single Cox and single AFT with L_1/2_regularization approaches for comparisons and for each procedure, the regularization parameters are tuned by 5-fold cross validation. All results in this article are averaged over 50 repeated times respectively.

**Fig. 4:**
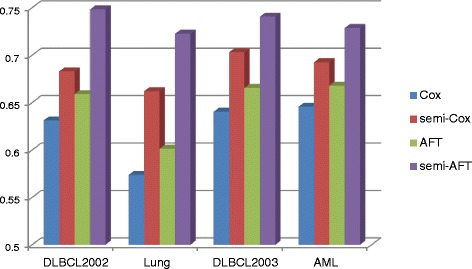
The CI obtained by the Cox and AFT models with and without semi-supervised learning approach for the four gene expression datasets

As show in Fig. 4, our proposed semi-supervised learning method can significantly increase the available sample size for classification model training. Especially, in Lung cancer dataset, the available samples increase from 27.91 to 94.19 %. For other three datasets, the available sample sizes also augment from 57.50, 69.56, 57.75 to 96.67, 96.73, 94.84 % respectively. Most censored data were accurately estimated by the AFT model using samples, which belong to the same genotype disease classes, and were sequentially classified into high-risk or low-risk classes by the Cox model respectively. In addition of that, just small part of the censored data cannot conjugate into the available samples because their imputed survival time based on the AFT model is smaller than their observed censored time. The reason may be the individual differences of the patients.

**Fig. 5:**
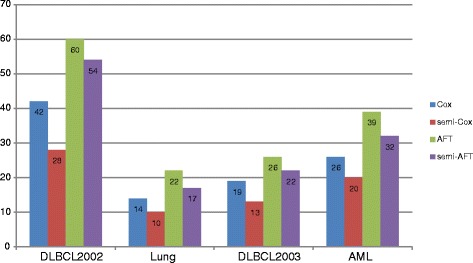
The number of genes selected by the Cox and AFT models with and without semi-supervised learning approach for the four gene expression datasets

**Fig. 6:**
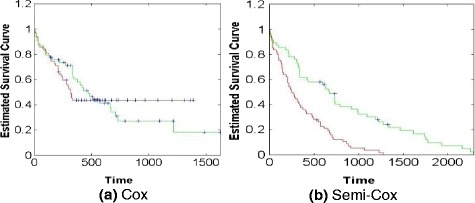
The survival curves of the Cox model with and without the semi-supervised learning method for AML dataset

The integrated brier score (IBS) and the concordance index (CI) measurements were used to evaluate the classification and prediction performance of Cox and AFT models in the semi-supervised learning approach. In the IBS measure, the lower value means the more accurate prediction result. As shown in Fig. 5, the values of IBS obtained by our semi-supervised learning model with L_1/2_ penalty were smaller than that obtained by the single Cox and AFT models. For example, in the Lung cancer dataset, the IBS values of the Cox and AFT models from 0.2164 and 0.2195 improve to 0.1259 and 0.1341 respectively in the semi-supervised learning approach. For the other gene expression datasets DLBCL2002, DLBCL2003 and AML, the IBS values of the Cox model improve 34, 45 and 26 %, and the IBS values of the AFT model improve 34, 36 and 28 % respectively. This means that our proposed semi-supervised learning approach can significantly improve the classification and prediction accuracy of the Cox and AFT models. In Fig. 6, the values of CI measure obtained by Cox and AFT with and without the semi-supervised learning approaches were given respectively. The CI values belong to the regain [0.5, 1] and its larger value means the more accurate prediction results. As shown in Fig 6, for the Lung cancer dataset, the CI values of the Cox and AFT models from 0.5738 and 0.6013 improve to 0.6620 and 0.7225 respectively in the semi-supervised learning approach. The improvement rate is higher than (0.6620-0.5738)/(0.5738-0.500) = 120 %. For the other gene expression datasets DLBCL2002, DLBCL2003 and AML, the CI values of the Cox models improve 39, 45 and 25 %, and the CI values of the AFT models improve 56, 45 and 36 % respectively. These also illustrated the semi-supervised learning method can significantly improve the accuracy of prediction in survival analysis with the high-dimensional and low sample size gene expression data.

**Fig. 7:**
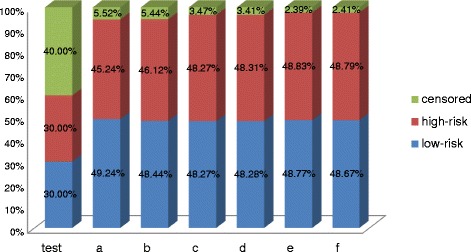
The percentage of different types of data processed by the semi-supervised learning model in simulated experiment

Figure 7 gives the number of genes selected by the L_1/2_regularized Cox and AFT models with and without the semi-supervised learning framework. The semi-Cox and semi-AFT selected less genes compared to the single Cox and the AFT model. For example, in the lung cancer dataset, the single Cox and single AFT models select 14 and 22 genes respectively. However, the Cox and AFT models just select 10 and 17 genes in semi-supervised learning model. Moreover, Combined the found in the Figs. 5 and 6, the prediction accuracy of Cox and AFT in the semi-supervised learning model was significantly improved using more relevant genes.

On the other hand, we find that for these all four gene expression datasets, the selected genes from Cox and AFT models are quite different and just small parts are overlapping. We think the reason may be that the regularized Cox model selects the relevant genes for low-risk and high-risk classification. Nerveless, the genes selected by the AFT model are high correlation for the survival time of patients. So these two models may select different genes, which have different biological function. Through our below analyses, we know that the genes selected by semi-supervised learning methods are significant relevant with the cancer.

**Fig. 8:**
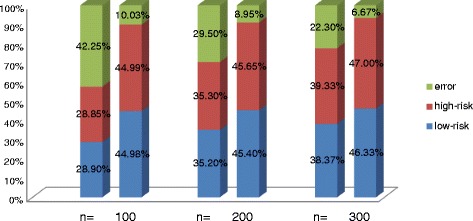
The percentage of correct and error classification obtained by our proposed semi-supervised learning model in simulated experiment

Figure 8 shows the survival curves of the Cox model with and without the semi-supervised learning method for AML dataset. The *x*-axis represents the survival days and the *y*-axis is the estimated survival probability. The green and read curves represent the changes of the survival probability for the “low-risk” and “high-risk” classes respectively. As show in Fig. 8a, these two curves intersect at the time point of 564 day, which means that the single Cox cannot efficiently classify and predict the survival rate of the patients using the AML dataset. On the contrary, in Fig. 8b, the survival probabilities of the “low-risk” and “high-risk” patients can be efficiently estimated by the semi-Cox model. For other three gene expression datasets, we also got the similar results, which are the classification performance of semi-Cox model significantly outperforms the single Cox model.

## Discussion

In this section, we introduce a brief biological discussion of the selected genes for the Lung cancer dataset to demonstrate the superiority of our proposed semi-supervised learning method. The number of selected genes by semi-supervised learning method is less than the single Cox and AFT model, but includes some genes which are significantly associated with cancer and cannot be selected by the two single Cox and AFT models, such as GDF15, ARHGDIB and PDGFRL. GDF15 belongs to the transforming growth factor-beta superfamily, and is one kind of bone morphogenetic proteins. It was showed that GDF15 can be seen as prognostication of cancer morbidity and mortality in men [31]. ARHGDIB is the member of the Rho (or ARH) protein family; it is involved in many different cell events such as cell secretion, proliferation. It is likely to impact on the cancer [32]. The role of PDGFRL is to encode a protein contains an important sequence which is similar to the ligand binding domain of platelet-derived growth factor receptor beta. Biological research has confirmed that this gene can affect the sporadic hepatocellular carcinomas. This suggests that this gene product may get the function of the tumour inhibition.

At the same time, the Cox and AFT models with and without semi-supervised learning method also selected some common genes. For example,the PTP4A2, TFAP2C, GSTT2. PTP4A2 is the member of the protein tyrosine phosphatase family, overexpression of PTP4A2 will confer a transformed phenotype in mammalian cells, which suggested its role in tumorigenic is [33]. TFAP2C can encode a protein contains a sequence-specific DNA-binding transcription factor which can activate some developmental genes [34]. GSTT2 is one kind of a member of a superfamily of proteins. It has been proved to play an important role in human carcinogenesis and shows that these genes are linked to cancer with a certain relationship [35].

Through the comparison of the biological analyses of the selected genes, we found the semi-supervised method based on Cox and AFT models with L_1/2_ regularization is a competitive method compared to single regularized Cox and AFT models.

## Conclusion

To overcome the limitations of fully unsupervised and fully supervised approaches for survival analysis in cancer research, we have developed a discriminative semi-supervised method based on Cox and AFT models with L_1/2_ regularization. This method combines the advantages of both Cox and AFT models, and overcome the dilemma in their applications. By comparison the results of Cox and AFT modes with and without the semi-supervised method in simulation experiment and real microarray datasets experiment with different regularizing method, we demonstrated that 1) the censored data could be employed after appropriate processing; 2) the semi-supervised classification improved prediction accuracy as compared to the state of the art single Cox model; 3) the gene selection performance gain improved with the increase number of available samples. Therefore, for clinical applications, where the goal is often to develop an accurate predicting test using fewer genes in order to control cost, the semi-supervised method based on Cox and AFT models with L_1/2_ regularization can be chosen to applied, it will be an efficient and accuracy method based on the high-dimensional and low-sample size data in cancer survival analysis.
